# Elevated IGF-1 levels are associated with accelerated bone age advancement during growth hormone therapy in children: a multicenter cohort study

**DOI:** 10.3389/fendo.2026.1843625

**Published:** 2026-08-19

**Authors:** Jungmin Ahn, Hwalrim Jeong, Junghwan Suh, Hyun Wook Chae

**Affiliations:** 1 Department of Pediatrics, Jeju National University Hospital, Jeju, Republic of Korea; 2 Department of Pediatrics, School of Medicine, Soonchunhyang University Cheonan Hospital, Cheonan, Republic of Korea; 3 Department of Pediatrics, Severance Children’s Hospital, Endocrine Research Institute, Yonsei University, Seoul, Republic of Korea

**Keywords:** bone age advancement, growth hormone therapy, idiopathic short stature, IGF-1 SDS, pediatric endocrinology, small for gestational age

## Abstract

**Background:**

Elevated insulin-like growth factor-1 (IGF-1) levels are associated with earlier pubertal onset and accelerated bone maturation. However, the effect of IGF-1 increases during growth hormone (GH) therapy on bone age (BA) advancement across different etiologies of short stature remains unclear.

**Methods:**

This multicenter observational study included 1,944 children with idiopathic growth hormone deficiency (IGHD), idiopathic short stature (ISS), or small for gestational age (SGA) enrolled in the LG Growth Study registry between 2020 and 2024. IGF-1 standard deviation score (SDS), auxological parameters, GH dose, and BA were assessed during GH therapy. BA advancement was defined as the first occurrence of a BA-to-chronological age ratio (BA/CA) >1 during follow-up. Cox proportional hazards regression models were used to evaluate the association between IGF-1 SDS and BA advancement, with additional multivariable adjustment for potential clinical confounders. Additional subgroup analyses according to age, sex, diagnosis, and GHD severity were performed. A sensitivity analysis using BA/CA >1.2 was also conducted.

**Results:**

Each 1-unit increase in IGF-1 SDS was associated with a higher risk of BA advancement in the overall cohort (hazard ratio [HR] 1.24, 95% confidence interval [CI] 1.15–1.33, p<0.001), with consistent associations in males and females. Compared with IGHD, children with ISS (HR 1.63, 95% CI 1.26–2.11) and SGA (HR 2.15, 95% CI 1.82–2.55) showed significantly increased risks of BA advancement. Additional analyses stratified by age, GHD severity, and pubertal initiation showed consistent findings. Sensitivity analysis using BA/CA >1.2 confirmed the association between IGF-1 SDS elevation and significant BA advancement.

**Conclusion:**

Increases in IGF-1 SDS during GH therapy are independently associated with accelerated bone maturation in children with short stature, particularly in those born SGA. These findings remained consistent across multiple subgroups and sensitivity analyses, supporting the importance of individualized GH dosing with careful monitoring of IGF-1 levels and skeletal maturation.

## Introduction

Several studies have demonstrated that elevated insulin-like growth factor-1 (IGF-1) levels are associated with an earlier onset and faster tempo of puberty in both sexes, suggesting a close relationship between circulating IGF-1 concentrations and pubertal timing (1). Experimental and clinical evidence indicates that the hypothalamic–pituitary–gonadal axis integrates a wide range of intrinsic and extrinsic signals related to growth, metabolism, and energy balance, among which insulin and IGF-1 play central roles within the central nervous system (2). Accordingly, insulin and IGF-1 have been extensively investigated for their regulatory effects on hypothalamic–pituitary function, pubertal development, and the nutritional modulation of reproduction.

Consistent with these observations, human studies have implicated the growth hormone (GH)–IGF-1 axis as a key determinant of pubertal timing. Reduced activity of this axis has been associated with delayed pubertal onset, whereas higher IGF-1 levels are linked to earlier pubertal progression (3). Children with growth hormone deficiency (GHD), therefore, frequently exhibit delayed puberty, which is largely attributable to insufficient IGF-1 secretion.

Within this context, GH replacement therapy plays a critical role in promoting skeletal maturation through pleiotropic mechanisms, including stimulation of linear growth, enhancement of bone mineral accrual, and support of peak bone mass acquisition (4). Although GH-induced increases in circulating IGF-1 are essential for achieving catch-up growth, excessive or rapid elevations in IGF-1 may accelerate growth plate maturation and potentially shorten the duration of linear growth. Despite this concern, evidence regarding the impact of IGF-1 elevation during GH therapy on bone age (BA) advancement remains limited.

Children born small for gestational age (SGA) and those with idiopathic short stature (ISS) represent distinct clinical populations frequently treated with GH and may demonstrate differential sensitivity to IGF-1–mediated skeletal maturation. However, whether GH-induced increases in IGF-1 differentially affects BA advancement among children with GHD, ISS, and SGA has not been clearly established.

Therefore, the objective of the present study was to evaluate the association between increases in IGF-1 standard deviation score (SDS) during GH therapy and the risk of BA advancement, defined as a BA-to-chronological age ratio (BA/CA) greater than 1, in children with GHD, ISS, and SGA.

## Methods

### Patients

**Figure 1:**
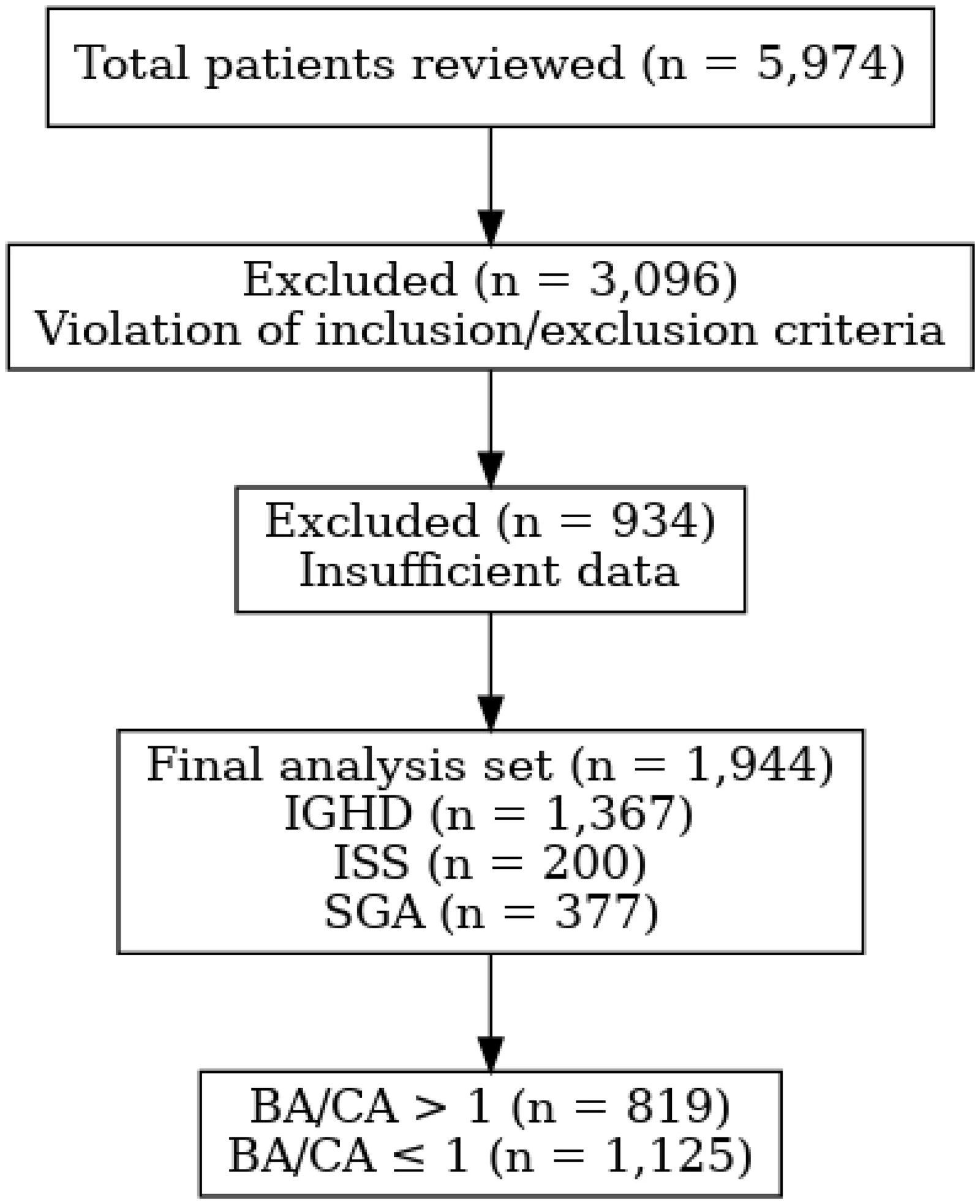
Flow diagram of patient selection and study design.

Patients were enrolled from the LG Growth Study (LGS), a multicenter observational registry designed to evaluate the long-term effectiveness and safety of GH therapy (Eutropin injection formulations; LG Chem Ltd., Korea) in children with GHD, ISS, or those born SGA (5). The registry was initiated on November 9, 2011, and data was accessed in May 2024. This analysis included 1,944 patients registered between 2020 and 2024 (**Figure 1**).

The diagnosis of idiopathic growth hormone deficiency (IGHD) followed the LGS etiology classification and was defined as height below −2.0 SDS and a peak GH response <10 ng/mL on at least two different GH stimulation tests (5, 6). Stimulation tests included clonidine, L-3,4-dihydroxyphenylalanine, glucagon, arginine, and insulin. All patients with IGHD had normal magnetic resonance imaging findings of the hypothalamic–pituitary region and no neurological symptoms.

To investigate whether the severity of GH deficiency influenced the association between IGF-1 SDS and BA advancement, IGHD patients were further stratified into complete GHD (peak GH <5 ng/mL) and partial GHD (peak GH 5–10 ng/mL) for subgroup analysis.

### Design and assessments

Clinical data including height, weight, BA, gestational age, birth weight, GH dose, serum IGF-1, IGF-binding protein-3, and thyroid function were collected at baseline and every 6 months thereafter.

IGF-1 SDS was calculated using age- and sex-specific Korean reference values (7). Height, weight, and body mass index SDS were derived from the 2017 Korean National Growth Charts (8). BA was assessed using the Greulich–Pyle method (9).

To evaluate potential differences related to age and pubertal development, additional analyses were performed according to chronological age groups (<10 years, 10–13 years, and ≥13 years). GH dose distributions were also compared among diagnostic groups to assess differences in treatment exposure.

### Outcome definition and statistical analysis

BA advancement was defined as a BA-to-chronological age ratio (BA/CA) >1.0.

For longitudinal analysis, BA/CA values were calculated using available follow-up measurements during GH therapy. IGF-1 SDS values obtained at corresponding time points were paired with BA/CA measurements.

The first occurrence of BA/CA >1.0 during follow-up was defined as the event. Patients who did not develop BA advancement during the observation period were censored at their last available follow-up visit.

Proportional hazards regression models were used to estimate hazard ratios (HRs) for BA advancement associated with IGF-1 SDS.

In the primary analysis, Model 1 was an unadjusted model, and Model 2 was adjusted for height SDS and weight SDS. Additional multivariable models were constructed with sequential adjustment for potential confounding factors, including age, sex, diagnosis, baseline height SDS, and baseline BA/CA.

Subgroup analyses were performed according to sex, age group, diagnostic category, and IGHD severity. Sensitivity analysis was performed using a stricter definition of BA advancement (BA/CA >1.2).

Kaplan–Meier survival analyses were used to compare time to BA advancement among diagnostic groups.

All statistical analyses were performed using SAS version 9.4 (SAS Institute Inc., Cary, NC, USA). A two-sided p value <0.05 was considered statistically significant.

### Ethnic statement

This study was approved by the Institutional Review Board of Jeju National University Hospital (IRB No. JEJUNUH 2023-08-021) and was conducted in accordance with the Declaration of Helsinki. Written informed consent was obtained from the parents or legal guardians of all participants, with assent from children when appropriate.

## Results

### Baseline clinical characteristics

Baseline clinical characteristics of the study population are summarized in **Table 1**.

**Table 1:** Baseline clinical characteristics of the study population.

	Male	Female	All	p-value^a^
Chronological age (years)				
n	1,146	798	1,944	
Mean ± SD	7.90 ± 2.99	7.76 ± 2.56	7.84 ± 2.82	0.8367
Median	7.19	7.5	7.33	Wilcoxon rank sum test
Q1, Q3	5.40, 10.36	5.67, 9.54	5.53, 9.96	
Min, Max	2.15, 16.25	2.24, 19.22	2.15, 19.22	
Bone age (years)				
n	1,146	798	1,944	
Mean ± SD	6.11 ± 3.04	6.43 ± 2.78	6.24 ± 2.94	0.0024
Median	5.3	6	5.7	Wilcoxon rank sum test
Q1, Q3	3.60, 8.00	4.00, 8.80	3.90, 8.50	
Min, Max	0.60, 14.50	0.80, 17.00	0.60, 17.00	
Height SDS				
n	1,146	798	1,944	
Mean ± SD	-2.51 ± 0.56	-2.59 ± 0.71	-2.54 ± 0.62	0.0741
Median	-2.41	-2.43	-2.43	Wilcoxon rank sum test
Q1, Q3	-2.75, -2.13	-2.82, -2.16	-2.77, -2.15	
Min, Max	-6.37, 1.05	-8.10, 0.78	-8.10, 1.05	
Weight SDS				
n	1,125	786	1,911	
Mean ± SD	-1.84 ± 0.99	-1.88 ± 1.06	-1.86 ± 1.02	0.827
Median	-1.88	-1.84	-1.86	Wilcoxon rank sum test
Q1, Q3	-2.43, -1.27	-2.45, -1.24	-2.44, -1.26	
Min, Max	-6.27, 3.38	-7.89, 1.27	-7.89, 3.38	
BMI SDS				
n	1,123	786	1,909	
Mean ± SD	-0.47 ± 1.12	-0.49 ± 1.12	-0.48 ± 1.11	0.9941
Median	-0.58	-0.58	-0.58	Wilcoxon rank sum test
Q1, Q3	-1.22, 0.23	-1.21, 0.22	-1.22, 0.22	
Min, Max	-5.25, 5.46	-4.65, 3.57	-5.25, 5.46	
IGF-1				
n	1,146	798	1,944	
Mean ± SD	136.43 ± 74.06	157.63 ± 78.37	145.13 ± 76.55	<0.0001
Median	124.78	145.75	133	Wilcoxon rank sum test
Q1, Q3	84.50, 177.59	101.00, 196.80	90.50, 185.00	
Min, Max	2.20, 607.00	4.75, 703.80	2.20, 703.80	
IGF-1 SDS				
n	1,146	798	1,944	
Mean ± SD	-0.67 ± 0.87	-0.79 ± 0.85	-0.72 ± 0.86	0.0015
Median	-0.75	-0.85	-0.8	Wilcoxon rank sum test
Q1, Q3	-1.20, -0.26	-1.33, -0.37	-1.25, -0.30	
Min, Max	-3.03, 5.32	-3.33, 3.54	-3.33, 5.32	

Values are presented as mean ± standard deviation. ^a^ P value for the difference between male and female groups. BA, bone age; BMI, body mass index; CA, chronological age; IGF-1, insulin-like growth factor-1; SDS, standard deviation score.

A total of 1,944 children (1,146 males and 798 females) were included in the analysis. The mean chronological age did not differ significantly between males (7.90 ± 2.99 years) and females (7.76 ± 2.56 years; p = 0.8367). However, females exhibited a significantly higher mean bone age compared with males (6.43 ± 2.78 vs. 6.11 ± 3.04 years; p = 0.0024).

Height SDS, weight SDS, and BMI SDS were comparable between sexes, with no statistically significant differences observed. Serum IGF-1 levels were higher in females; however, IGF-1 SDS was significantly lower in females compared with males (−0.79 ± 0.85 vs. −0.67 ± 0.87; p = 0.0015).

### Association between IGF-1 SDS and bone age advancement

The association between IGF-1 SDS and the risk of BA advancement, defined as BA/CA >1.0, was evaluated using Cox proportional hazards regression models (**Table 2**).

**Table 2:** Hazard ratios (HR) and 95% CI between IGF-1 SDS and BA/CA>1 for all patients.

	Total		Male		Female	
	N	HR [95% CI]	N	HR [95% CI]	N	HR [95% CI]
Model 1	1,910	1.26 [1.17-1.35]	1,124	1.26 [1.14-1.40]	786	1.31 [1.19-1.44]
Model 2	1,910	1.24 [1.15-1.33]	1,124	1.27 [1.14-1.41]	786	1.23 [1.12-1.35]

Model 1: unadjusted. Model 2: adjusted for height SDS and weight SDS.

In the overall cohort, each 1-unit increase in IGF-1 SDS was significantly associated with an increased risk of BA advancement. This association remained significant after adjustment for height SDS and weight SDS (HR 1.24, 95% CI 1.15–1.33, p<0.001). Similar associations were observed in both male and female patients.

Additional analyses were performed to confirm the stability of these findings. When a stricter definition of BA advancement (BA/CA >1.2) was applied, IGF-1 SDS remained significantly associated with advanced skeletal maturation (HR 1.46, 95% CI 1.23–1.72, p<0.0001). Subgroup analyses according to sex and diagnostic group showed consistent trends, with stronger associations observed in females and children born SGA (**Supplementary Table 1**).

In sequential multivariable models adjusting for potential confounding factors, including age, sex, diagnosis, baseline height SDS, and baseline BA/CA, IGF-1 SDS remained independently associated with BA advancement (fully adjusted model: HR 1.20, 95% CI 1.11–1.28, p<0.0001; **Supplementary Table 3A**).

Age-stratified analyses showed that increased IGF-1 SDS was significantly associated with BA advancement in children aged <10 years and 10–13 years (**Supplementary Table 3B**).

### Diagnostic group–specific risk and Kaplan–Meier analysis

Hazard ratios for BA advancement according to diagnostic group are presented in **Table 3**.

**Table 3:** Hazard ratios (HRs) and 95% confidence intervals (CIs) for the association between IGF-1 SDS and BA/CA > 1 in patients with SGA and ISS, using IGHD as the reference group.

	Total		Male		Female	
	N	HR [95% CI]	N	HR [95% CI]	N	HR [95% CI]
Model 1	1,910		1,124		786	
IGF-1 SDS		1.24 [1.15-1.33]		1.25 [1.13-1.39]		1.27 [1.15-1.40]
Diagnosis^a^						
ISS		1.47 [1.14-1.89]		1.34 [0.89-2.02]		1.34 [0.97-1.86]
SGA		1.89 [1.60-2.23]		1.95 [1.53-2.48]		1.67 [1.33-2.10]
Model 2	1,910		1,124		786	
IGF-1 SDS		1.21 [1.12-1.3]		1.26 [1.13-1.40]		1.18 [1.07-1.30]
Diagnosis^a^						
ISS		1.63 [1.26-2.11]		1.47 [0.97-2.22]		1.51 [1.09-2.10]
SGA		2.15 [1.82-2.55]		2.27 [1.77-2.91]		1.85 [1.47-2.34]

^a^ reference: IGHD.

Compared with children with IGHD, those with ISS and SGA showed significantly increased risks of BA advancement. The highest risk was observed in the SGA group, followed by the ISS group.

**Figure 2:**
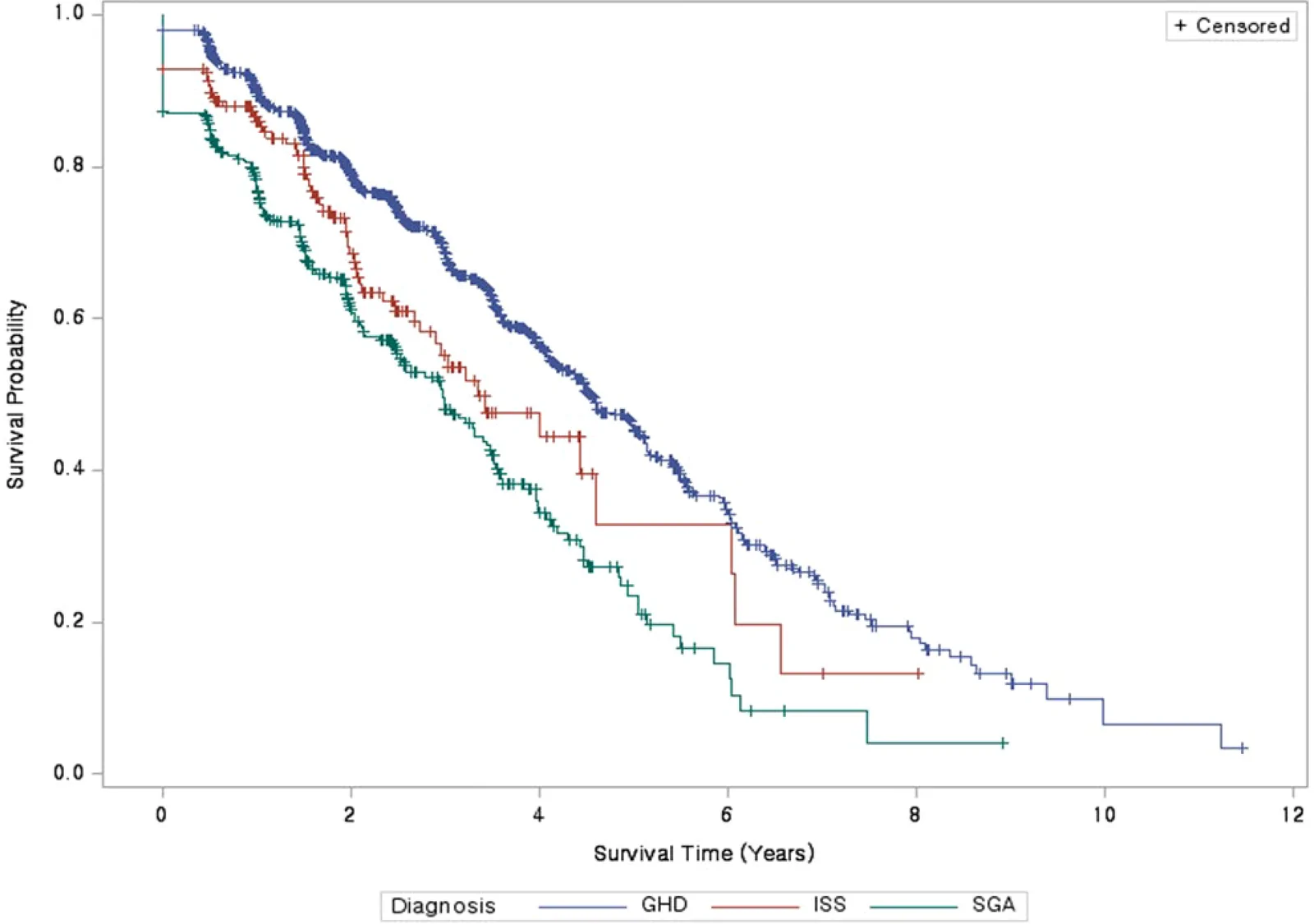
Kaplan–Meier curves for time to bone age advancement. (BA/CA > 1) according to diagnostic group. Blue, IGHD; red, ISS; green, SGA.

Kaplan–Meier curves demonstrated differences in time to BA advancement among diagnostic groups (**Figure 2**). Children born SGA showed the earliest progression to BA/CA >1.0, followed by those with ISS, whereas children with IGHD showed the slowest progression.

Additional diagnostic subgroup analyses further supported these findings. Among patients with IGHD, stratification according to GH deficiency severity (complete GHD, peak GH <5 ng/mL; partial GHD, peak GH 5–10 ng/mL) showed comparable associations between IGF-1 SDS and BA advancement (**Supplementary Table 2**).

GH dose distributions across diagnostic groups are presented in **Supplementary Table 4**.

## Discussion

In this large multicenter observational cohort, elevated IGF-1 SDS during growth hormone (GH) therapy was independently associated with an increased risk of bone age (BA) advancement across diagnostic groups. Importantly, this association remained significant after sequential adjustment for potential confounding factors, including age, sex, diagnosis, baseline height SDS, and baseline BA/CA, and was also confirmed using a stricter definition of BA advancement (BA/CA >1.2). These findings support the robustness of the association between IGF-1 elevation and accelerated skeletal maturation during GH therapy. The association remained evident and appeared stronger when the stricter BA/CA >1.2 criterion was applied. Although this subgroup contained substantially fewer events, the findings further support the robustness of the association between elevated IGF-1 SDS and accelerated skeletal maturation.

The magnitude of this association differed according to the underlying growth disorder, with the highest susceptibility observed in children born small for gestational age (SGA), followed by those with idiopathic short stature (ISS), compared with children with idiopathic growth hormone deficiency (IGHD). These findings suggest that GH-induced increases in IGF-1 may exert differential effects on skeletal maturation depending on individual growth etiology and biological sensitivity to GH therapy, extending previous observations linking circulating IGF-1 levels with pubertal timing and skeletal development (1–4).

The GH–IGF-1 axis is a central regulator of postnatal skeletal growth, acting through both endocrine and local mechanisms at the epiphyseal growth plate (9–12). IGF-1 stimulates chondrocyte proliferation within the proliferative zone and promotes hypertrophic differentiation, thereby accelerating longitudinal bone growth (11–14). Although restoration of IGF-1 signaling is essential for achieving catch-up growth during GH therapy, excessive or sustained IGF-1 exposure may accelerate growth plate maturation, shorten the remaining period of linear growth, and potentially contribute to earlier epiphyseal fusion (15–17).

At the molecular level, IGF-1 activates intracellular signaling pathways, including the phosphoinositide 3-kinase (PI3K)/Akt and mitogen-activated protein kinase (MAPK) pathways, which regulate chondrocyte proliferation, hypertrophy, and survival (18–20). These signaling pathways interact with local paracrine regulators within the growth plate microenvironment and systemic metabolic signals, suggesting that persistent elevation of circulating IGF-1 may enhance growth plate activity beyond physiological requirements (19–22). Experimental studies have also suggested that prolonged activation of growth-promoting pathways may contribute to accelerated growth plate senescence, providing a potential biological explanation for the association between higher IGF-1 SDS and BA advancement observed in our cohort (16, 17, 21).

Children born SGA are known to exhibit distinct growth patterns characterized by early catch-up growth, altered metabolic programming, and variable responsiveness to GH and IGF-1 (23–28). In the present study, Kaplan–Meier analysis demonstrated more rapid progression to BA/CA >1.0 in SGA patients than in ISS or IGHD patients. These findings suggest that children born with SGA may have increased skeletal sensitivity to IGF-1-mediated effects and therefore require careful monitoring of BA progression during GH treatment.

ISS represents a heterogeneous population of children with short stature and preserved GH secretion, in whom growth outcomes may be influenced by genetic background, familial growth patterns, and individual sensitivity to GH therapy (29–31). In our study, ISS patients showed an intermediate risk of BA advancement between IGHD and SGA. These findings suggest that variability in growth plate responsiveness to IGF-1 may contribute to differences in skeletal maturation during GH therapy among ISS patients (31, 32). These findings suggest that variability in growth plate responsiveness to IGF-1 may contribute to differences in skeletal maturation during GH therapy among ISS patients. Previous studies have suggested that GH responsiveness may be reduced during puberty in some patients with ISS because of rapid bone age advancement and the consequent reduction in remaining growth potential. In the present study, the number of pubertal participants was limited, which may explain why this effect was not clearly demonstrated (29, 32).

Because IGHD includes patients with varying degrees of GH deficiency, we further evaluated whether IGF-1-related BA advancement differed according to GHD severity. Subgroup analyses demonstrated that increased IGF-1 SDS was significantly associated with BA advancement in both complete and partial GHD groups. These findings suggest that the relationship between IGF-1 exposure and skeletal maturation is not restricted to a specific severity of GH deficiency but may reflect individual responsiveness to achieved IGF-1 levels during treatment.

GH dose is an important determinant of IGF-1 elevation and growth response during therapy. However, additional adjustment for average GH dose did not substantially alter the association between IGF-1 SDS and BA advancement in the present study. This finding suggests that achieved IGF-1 SDS may represent an integrated marker reflecting not only GH dose exposure but also individual GH sensitivity and biological response. Therefore, monitoring IGF-1 SDS together with auxological parameters may provide additional information beyond GH dose alone.

Age-stratified analyses further demonstrated significant associations between IGF-1 SDS and BA advancement in children aged <10 years and 10–13 years. The association did not reach statistical significance in patients aged >13 years, which may be partly explained by the smaller number of patients and BA advancement events in this subgroup, as well as more advanced skeletal maturation at older ages. During the peripubertal period, endogenous sex steroids interact with the GH–IGF-1 axis and may further accelerate growth plate maturation (18–22, 33). Therefore, careful interpretation of IGF-1 levels in relation to pubertal stage and skeletal maturation is important.

Clinically, these findings emphasize the importance of individualized GH treatment strategies. Maintaining IGF-1 concentrations within age- and sex-adjusted reference ranges may help balance short-term catch-up growth against the potential risk of excessive skeletal advancement and compromised remaining growth potential (34–36). Regular assessment of BA together with IGF-1 monitoring may allow earlier identification of patients showing disproportionate skeletal maturation and enable timely modification of treatment strategies.

The strengths of this study include its large sample size, multicenter registry design, and use of time-to-event analyses to evaluate longitudinal changes in BA progression during GH therapy. However, several limitations should be acknowledged. First, the observational nature of the study prevents determination of causal relationships. Second, detailed pubertal parameters, including standardized Tanner staging, serial sex hormone measurements, and gonadal imaging data, were not uniformly available in this registry. Because sex steroids interact with IGF-1 in regulating skeletal maturation, residual confounding related to pubertal status cannot be completely excluded. Third, parental height and familial growth patterns were not consistently available, which may be particularly relevant for ISS patients. Finally, laboratory measurements were performed across multiple centers, and potential inter-assay variability should be considered despite standardized clinical protocols.

In conclusion, increased IGF-1 SDS during GH therapy was independently associated with a higher risk of BA advancement in children with IGHD, ISS, and SGA, with the greatest susceptibility observed among children born SGA. These findings support individualized GH treatment approaches integrating growth response, GH dose, IGF-1 SDS monitoring, and skeletal maturation assessment to optimize long-term growth outcomes while minimizing excessive acceleration of skeletal development.

## Data Availability

The raw data supporting the conclusions of this article will be made available by the authors, without undue reservation.
