# Supplementary material for: Elevated IGF-1 levels are associated with accelerated bone age advancement during growth hormone therapy in children: a multicenter cohort study
Source: Front Endocrinol (Lausanne). 2026 Aug 19;17:1843625. doi: 10.3389/fendo.2026.1843625 (PMC13533664; doi:10.3389/fendo.2026.1843625)
Supplement: Supplementary file 1 [file Table1.docx]

| Subgroup | N | Events, BA/CA >1.2 | Event rate (%) | HR per 1-SDS increase in IGF-1 SDS | 95% CI | p-value |
| --- | --- | --- | --- | --- | --- | --- |
| Overall | 1,943 | 76 | 3.9 | 1.46 | [1.23-1.72] | <0.0001 |
| Sex |  |  |  |  |  |  |
| Male | 1,145 | 24 | 2.1 | 1.30 | [0.98-1.71] | 0.0651 |
| Female | 798 | 52 | 6.5 | 1.49 | [1.15-1.90] | 0.0015 |
| Diagnosis |  |  |  |  |  |  |
| IGHD | 1,367 | 30 | 2.2 | 1.16 | [0.86-1.55] | 0.3242 |
| ISS | 200 | 6 | 3.0 | 4.09 | [1.07-15.61] | 0.0392 |
| SGA | 376 | 40 | 10.6 | 1.8 | [1.38-2.35] | <0.0001 |

**Supplementary Table 1.** Sensitivity analysis using BA/CA >1.2 by sex

Model, crude (IGF-1 SDS) adjusted by Age + diagnosis + Baseline Height SDS + Bone age/ Chronological age (Screening)

**Supplementary Table 2**. Subgroup analysis using BA/CA >1.0 in complete and partial IGHD

| Subgroup (peak GH <5 vs 5–10) | N | Events, BA/CA >1.0 | Event rate (%) | HR per 1-SDS increase in IGF-1 SDS | 95% CI | p-value |
| --- | --- | --- | --- | --- | --- | --- |
| Complete GHD, peak GH <5 ng/mL^1^ | 403 | 164 | 40.7 | 1.35 | [1.14-1.59] | 0.0005 |
| Partial GHD, peak GH 5–10 ng/mL^2^ | 998 | 400 | 40.1 | 1.20 | [1.07-1.35] | 0.0024 |

Model, crude (IGF-1 SDS) adjusted by Age + Sex + Baseline Height SDS + Bone age/ Chronological age (Screening)

**Supplementary Table 3A**. Crude and adjusted models of IGF-1 SDS and BA advancement

| N | Events, BA/CA >1.0 | Event rate (%) | HR per 1-SDS increase in IGF-1 SDS | HR per 1-SDS increase in IGF-1 SDS | p-value |
| --- | --- | --- | --- | --- | --- |
| 1,943 | 819 | 42.2 | 1.20 | [1.11-1.28] | <0.0001 |

Model, crude (IGF-1 SDS) adjusted by Age + Sex + Baseline Height SDS + Bone age/ Chronological age (Screening)

**Supplementary Table 3B.** Age-stratified analysis of the association between IGF-1 SDS and bone age advancement

| Age group | N | Events, BA/CA >1.0 | Event rate (%) | HR per 1-SDS increase in IGF-1 SDS | 95% CI | p-value |
| --- | --- | --- | --- | --- | --- | --- |
| <10 years | 1,462 | 672 | 46.0 | 1.13 | [1.04-1.21] | 0.0026 |
| 10-13 years | 403 | 141 | 35.0 | 1.68 | [1.27-2.23] | 0.0003 |
| >13 years | 78 | 6 | 7.7 | 2.62 | [0.34-20.00] | 0.3529 |

Model, crude (IGF-1 SDS) adjusted by Age + Sex + Baseline Height SDS + Bone age/ Chronological age (Screening)

**Supplementary Table 4**. Effect of GH dose adjustment on the association between IGF-1 SDS and BA/CA > 1.0

| Subgroup | N | Model | Variable in Model | HR | 95% CI | p-value |
| --- | --- | --- | --- | --- | --- | --- |
| Overall | 1,943 | Model 4 | IGF-1 SDS | 1.20 | [1.11-1.28] | <0.0001 |
|  |  | Model 4 + GH dose | IGF-1 SDS | 1.21 | [1.13-1.30] | <0.0001 |
|  |  |  | Average GH dose | 0.16 | [0.07-0.38] | <0.0001 |
| IGHD | 1,367 | Model 4 | IGF-1 SDS | 1.18 | [1.08-1.28] | 0.0002 |
|  |  | Model 4 + GH dose | IGF-1 SDS | 1.19 | [1.10-1.30] | <0.0001 |
|  |  |  | Average GH dose | 0.15 | [0.06-0.39] | <0.0001 |
| ISS | 200 | Model 4 | IGF-1 SDS | 1.11 | [0.76-1.62] | 0.5919 |
|  |  | Model 4 + GH dose | IGF-1 SDS | 1.10 | [0.76-1.61] | 0.6080 |
|  |  |  | Average GH dose | 0.17 | [0.00-247.47] | 0.6331 |
| SGA | 376 | Model 4 | IGF-1 SDS | 1.23 | [1.06-1.43] | 0.0073 |
|  |  | Model 4 + GH dose | IGF-1 SDS | 1.23 | [1.06-1.43] | 0.0077 |
|  |  |  | Average GH dose | 0.22 | [0.02-3.40] | 0.2807 |

Model 4: Adjusted by Age, Sex, Diagnosis, Baseline Height SDS, and Bone age/Chronological age (Screening)

Model 4 + GH dose: Adjusted by Model 4 + Average GH dose

Average GH dose: Average GH dose during full treatment period (mg/kg/week)
